# Quantitative myocardial first-pass cardiovascular magnetic resonance perfusion imaging using hyperpolarized [1-^13^C] pyruvate

**DOI:** 10.1186/s12968-018-0495-2

**Published:** 2018-11-12

**Authors:** Maximilian Fuetterer, Julia Busch, Julia Traechtler, Patrick Wespi, Sophie M. Peereboom, Mareike Sauer, Miriam Lipiski, Thea Fleischmann, Nikola Cesarovic, Christian T. Stoeck, Sebastian Kozerke

**Affiliations:** 1 Institute for Biomedical Engineering, University and ETH Zurich, Gloriastrasse, 35 8092 Zurich, Switzerland; 2 Division of Surgical Research, University Hospital Zurich, Sternwartstrasse, 14 8091 Zurich, Switzerland

**Keywords:** Perfusion imaging, Myocardial blood flow, Perfusion quantification, Hyperpolarization, 13C pyruvate

## Abstract

**Background:**

The feasibility of absolute myocardial blood flow quantification and suitability of hyperpolarized [1-^13^C] pyruvate as contrast agent for first-pass cardiovascular magnetic resonance (CMR) perfusion measurements are investigated with simulations and demonstrated in vivo in a swine model.

**Methods:**

A versatile simulation framework for hyperpolarized CMR subject to physical, physiological and technical constraints was developed and applied to investigate experimental conditions for accurate perfusion CMR with hyperpolarized [1-^13^C] pyruvate. Absolute and semi-quantitative perfusion indices were analyzed with respect to experimental parameter variations and different signal-to-noise ratio (SNR) levels. Absolute myocardial blood flow quantification was implemented with an iterative deconvolution approach based on Fermi functions. To demonstrate in vivo feasibility, velocity-selective excitation with an echo-planar imaging readout was used to acquire dynamic myocardial stress perfusion images in four healthy swine. Arterial input functions were extracted from an additional image slice with conventional excitation that was acquired within the same heartbeat.

**Results:**

Simulations suggest that obtainable SNR and B_0_ inhomogeneity in vivo are sufficient for the determination of absolute and semi-quantitative perfusion with ≤25% error. It is shown that for expected metabolic conversion rates, metabolic conversion of pyruvate can be neglected over the short duration of acquisition in first-pass perfusion CMR. In vivo measurements suggest that absolute myocardial blood flow quantification using hyperpolarized [1-^13^C] pyruvate is feasible with an intra-myocardial variability comparable to semi-quantitative perfusion indices.

**Conclusion:**

The feasibility of quantitative hyperpolarized first-pass perfusion CMR using [1-^13^C] pyruvate has been investigated in simulations and demonstrated in swine. Using an approved and metabolically active compound is envisioned to increase the value of hyperpolarized perfusion CMR in patients.

## Background

Qualitative and semi-quantitative myocardial perfusion cardiovascular magnetic resonance (CMR) imaging using gadolinium based contrast agents is a clinically established modality for diagnosing coronary artery disease and ischemia [1, 2]. To reduce operator dependence during analysis and to enable the assessment of triple-vessel disease, microvascular obstruction and other conditions that present themselves by global or diffuse perfusion deficits, absolute myocardial blood flow (MBF) quantification is promoted [3, 4]. Absolute MBF quantification requires an accurate estimation of the impulse response function (IRF), which links the concentration of an arterial input function (AIF) and the response concentration in the myocardium, and scales linearly with the MBF. In conventional first-pass perfusion CMR bolus administration of chelated gadolinium-based contrast agents is used to accelerate spin-lattice relaxation subject to contrast agent concentration. The resulting dynamic contrast enhancement (DCE) provides contrast between ischemic and normally perfused myocardial tissue [5, 6] with commonly used saturation recovery sequences [7]. However, determination of contrast agent concentrations from measured signal intensities is complicated by the non-linearity of spin-lattice relaxation and saturation effects during acquisition. To address these issues, dual-bolus [5, 8] or variable saturation delay [9] approaches have been proposed at the cost of additional bolus administrations and/or non-trivial correction steps.

Dissolution dynamic nuclear polarization enables the production of highly polarized endogenous ^13^C labelled molecules in solution with > 10,000-fold enhanced signal relative to thermal equilibrium [10]. We have previously demonstrated the feasibility of hyperpolarized first-pass perfusion CMR using ^13^C urea in porcine models [11]. Due to the lack of thermal background signal, hyperpolarized contrast agents benefit from high contrast to noise ratio (CNR) compared with gadolinium-based measurements, as well as linear dependency of signal intensity with respect to concentration, which makes them promising alternatives for absolute MBF quantification. To avoid unwanted effects from metabolic conversion, hyperpolarized perfusion imaging has so far been limited to ^13^C urea and other metabolically inert substrates such as HP001 [12], tert-butanol [13] or α-trideuteromethyl [^15^N] glutamine [14].

Pyruvate is metabolically active and a vital intermediate in the process of glycolysis, where it is converted into lactate, carbon dioxide (C0_2_)/ bicarbonate and alanine. As a hyperpolarized substrate, [1-^13^C] pyruvate has been widely used to probe the glycolytic pathway in various organs of the body, including the heart [15–17]. To better understand the pathological alterations in metabolism and to improve diagnostic value, quantification of the metabolic conversion rates in form of kinetic modelling has been applied in tumours [18, 19] and the isolated and in-vivo rat heart [20, 21]. This approach however requires accurate knowledge of the bolus input in the target organ for quantification and discrimination of metabolic and perfusion related signal variations. To this end tailored blood suppressing sequences and co-polarized administration of [^13^C] urea have been proposed [22, 23].

With recent progress in the translation of hyperpolarized [1-^13^C] pyruvate into clinical application [24] and first successful administrations in the human heart [25], the need for accurate perfusion assessment without additional measurements has been highlighted. Extensive alterations and global as well as diffuse deficits are only detectable by quantitative assessment of metabolic processes with kinetic modelling and knowledge of the underlying perfusion. As regulatory approval of hyperpolarized substrates for human administration is limited to [1-^13^C] pyruvate for the foreseeable future, previously proposed perfusion substrates are currently not suited for clinical trials.

In this work we therefore explore the feasibility and accuracy of absolute and semi-quantitative perfusion CMR using [1-^13^C] pyruvate. By administration of a sharp contrast agent bolus and using velocity-selective excitation [11], we show that myocardial perfusion can rapidly be assessed before metabolic conversion significantly alters myocardial pyruvate signal intensities. Extensive simulations including MBF, metabolic conversion and physical imaging constraints inform on the limitations and requirements for accurate determination of absolute and semi-quantitative perfusion indices. In vivo feasibility and absolute MBF quantification is demonstrated in a swine model.

## Methods

### Simulation framework

**Fig. 1:**
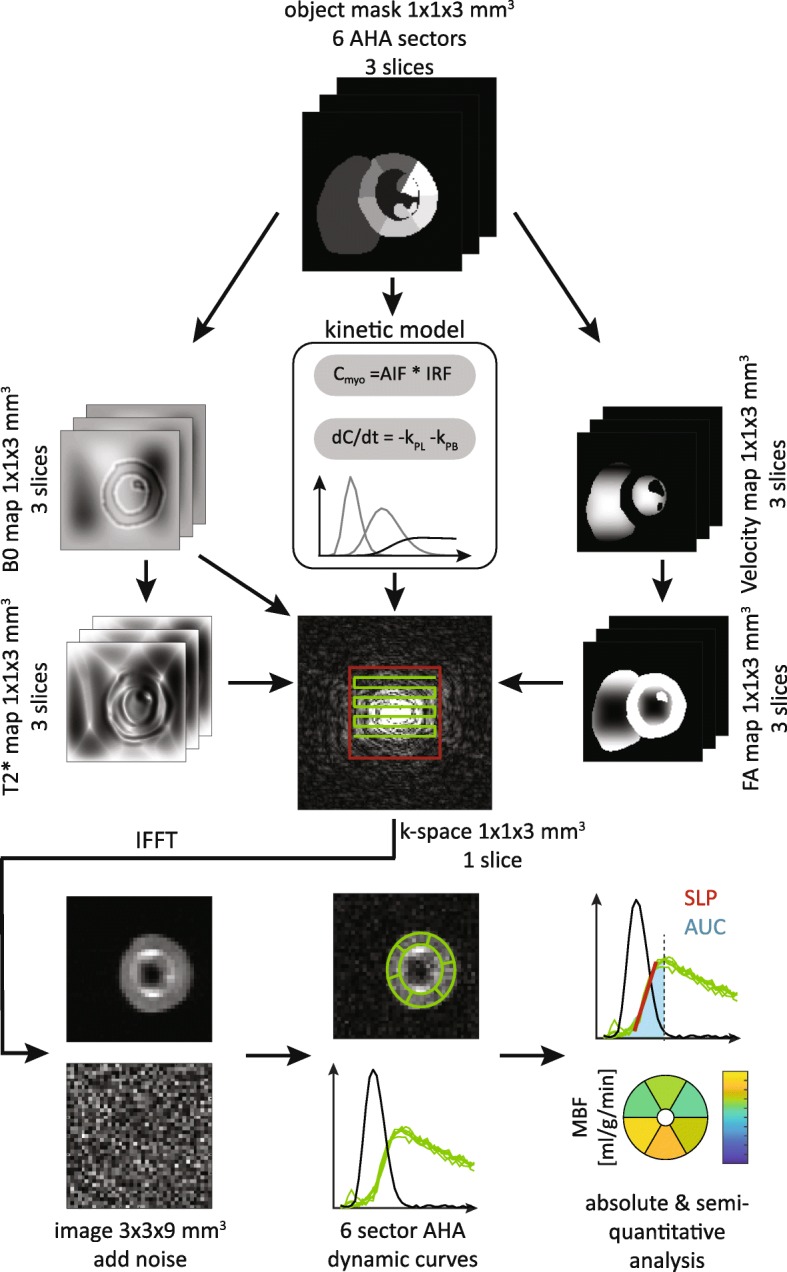
Simulation pipeline overview. Dynamic cardiac perfusion image series are generated from a six sector MRXCAT model in short axis view. Concentration dynamics are calculated from a convolutional perfusion model and a first order kinetic model for metabolic conversion. Spatial variation of B_0_ inhomogeneities, T2* relaxation and excitation profiles are included as synthetic maps. K-space sampling is implemented for arbitrary trajectories with phase evolutions for each sampling time point. Three high resolution slices are down-sampled into one image slice. Perfusion curves are extracted from noisy images at eight different signal-to-noise (SNR) levels with 20 realizations each

A high resolution computational model of a short axis view of the heart was generated from the MRXCAT phantom [26] at a field-of-view (FOV) of 120 × 120 mm^2^. To account for limited sampling resolution and intra-slice effects, the initial phantom consists of three slices of 3 mm thickness with an in-plane resolution of 1 × 1 mm^2^. A total of eight anatomical compartments were defined based on the mid left ventricular (LV) slice of the American Heart Association (AHA) segmentation [27]: right ventricular (RV) blood pool, LV blood pool and six myocardial segments. K-space sampling subject to several physical and physiological effects were then modeled as illustrated in Fig. 1. The full signal model for the presented simulation framework for sampling one 2D slice consisting of 3 isochromat sub-slices is given by:

1 $$ S\left(\overset{\rightharpoonup }{k},{t}_d\right)=\sum \limits_{\overset{\rightharpoonup }{r}}\sum \limits_{t_s}{e}^{j\overset{\rightharpoonup }{k}\left({t}_s\right)\overset{\rightharpoonup }{r}}\cdot \sin \left(\alpha \left(\overset{\rightharpoonup }{r}\right)\right)\cdot \sum \limits_{m=1}^M\left[{C}_m\left(\overset{\rightharpoonup }{r},{t}_d\right)\cdot {e}^{j2\pi {f}_m{t}_s}\cdot {e}^{-{t}_d/{T}_{1,m}}\cdot {e}^{-{t}_s/{T}_{2,m}^{\ast}\left(\overset{\rightharpoonup }{r}\right)}\right]\cdot {e}^{j2\pi \overline{\gamma}{dB}_o\left(\overset{\rightharpoonup }{r}\right){t}_s} $$

where $\overset{\rightharpoonup }{r}={\left(x,y,z\right)}^T$ and $\overset{\rightharpoonup }{k}={\left({k}_x,{k}_y,{k}_z\right)}^T$ denote the spatial coordinates in image space and k-space respectively, *t*_*d*_ the time point in the dynamic series, *α* the spatially varying flip angle, *C*_*m*_, *f*_*m*_, *T*_1, *m*_ the concentration, frequency offset and longitudinal relaxation time of metabolite *m*, and *B*_0_, ${T}_{2,m}^{\ast }$ the off-resonance and apparent transverse relaxation times. The time dependent k-space trajectory*k*(*t*_*s*_) with sampling time points *t*_*s*_ enables k-space sampling along arbitrary trajectories and at arbitrary resolutions considering dephasing effects during the readout.

Dynamic pyruvate concentrations were simulated in a two-step process: Firstly, MBF was modeled by convolution of a generic AIF from the MRXCAT model in the LV with a Fermi function shaped IRF:

2 $$ {\displaystyle \begin{array}{l}{C}_{myo}(t)= IRF(t)\ast {AIF}_{LV}(t)\\ {} IRF(t)= MBF\cdot \frac{1+\beta }{1+\beta {e}^{\alpha t}}\end{array}} $$

with shape parameters *α*, *β* = 0.25, temporal sampling interval 0.5 s (heart rate 120 bpm) and varying MBF. In a second step, a forward kinetic model was employed to account for metabolic conversion of pyruvate (Pyr) into lactate (Lac) and bicarbonate (Bic):

3 $$ \frac{d}{dt}\left[\begin{array}{c}{C}_{Pyr}(t)\\ {}{C}_{Lac}(t)\\ {}{C}_{Bic}(t)\end{array}\right]=\left[\begin{array}{ccc}-{k}_{PL}-{k}_{PB}& {k}_{LP}& 0\\ {}{k}_{PL}& -{k}_{LP}& 0\\ {}{k}_{PB}& 0& 0\end{array}\right]\cdot \left[\begin{array}{c}{C}_{Pyr}(t)\\ {}{C}_{Lac}(t)\\ {}{C}_{Bic}(t)\end{array}\right]+\left[\begin{array}{c}\frac{d}{dt}{C}_{myo}(t)\\ {}0\\ {}0\end{array}\right] $$

Where *k*_*PL*_, *k*_*PB*_ denote the kinetic forward conversion rates from pyruvate into lactate and bicarbonate, respectively. Reverse conversion from lactate to pyruvate was assumed negligible (*k*_*LP*_ = 0).

Off-resonances were simulated as a high resolution B_0_ map generated from a superposition of a three-dimensional second order polynomial and a filtered replica of the anatomical model. A convolution of the anatomical object with a Laplacian of Gaussian filter (width 8 mm, σ = 2 mm) was used to create an object that mimics strong susceptibility gradients at tissue interfaces as described in [28]. The combined B_0_ map was then scaled to the desired maximum in-plane and through-slice off-resonance.

4 $$ {B}_0\left(x,y,z\right)= LoG\left(x,y\right)\ast {Myo}_z\left(x,y\right)+{f}_{poly}\left(x,y,z\right)+{B}_{dz}(z) $$

where *LoG* denotes the filter kernel, *Myo*_*z*_ the myocardial object mask, *f*_*poly*_ the 3D 2nd order polynomial and *B*_*dz*_ the B_0_ through-slice gradient.

Spatially varying T2* relaxation was calculated from the first order derivative of the B_0_ map along phase and measurement direction using a 2D Gaussian kernel (σ = 3):

5 $$ \frac{1}{T_{2,m}^{\ast}\left(x,y\right)}=\frac{1}{T_{2,m}}+{G}_{\Delta x}\ast {B}_0\left(x,y\right)+{G}_{\Delta y}\ast {B}_0\left(x,y\right) $$

With *T*_2, *m*_ the native *T*_2_ of metabolite *m*, and *G*_Δ*x*_, *G*_Δ*y*_ the 2D Gaussian kernels along x and y, respectively.

Velocity-selective excitation [11] was simulated assuming a parabolic velocity distribution in the RV and LV according to:

6 $$ v\left(x,y\right)={v}_{\mathrm{max}}\sqrt{{\left(\frac{1}{2w}{\left(x-{x}_c\right)}^2\right)}^2+{\left(\frac{1}{2w}{\left(y-{y}_c\right)}^2\right)}^2} $$

where *w* denotes the width of the distribution and (*x*_*c*_, *y*_*c*_) the center of mass of the respective blood compartment. Based on the velocity distribution, the effective spatially dependent flip angle of the selective excitation is given by:

7 $$ {\alpha}_{eff}\left(x,y\right)=\alpha \cdot \left|\sin \left(\frac{v_{enc}-v\left(x,y\right)}{v_{enc}}\frac{\pi }{2}\right)\right| $$

where *α* denotes the nominal flip angle and *v*_*enc*_ the encoding velocity of the excitation.

An echo planar imaging (EPI) trajectory for k-space sampling was generated from gradient waveforms with hardware limits in accordance to in vivo experiments on a clinical 3 T system (slew rate 195 T/m/s, amplitude 30 mT/m). Using Partial Fourier acquisition (factor 0.65) the effective number of profiles was reduced from 39 to 25, resulting in a readout duration of 35 ms for the target in-plane resolution of 3 × 3 mm^2^ and a 120 × 120 mm^2^ FOV.

Computationally the proposed model as described by Equation (1) is challenging, due to the inclusion of the phase evolution stemming from B_0_ inhomogeneities. Consequently, the resulting phase maps need to be calculated for each k-space sampling time point *t*_*s*_, which significantly increases the memory requirements. The simulation of a dynamic k-space series with 40 dynamics took approximately 5 min on a workstation equipped with a hexa-core Xeon X5670 processor and 256 GB of memory.

In order to assess the sensitivity and to inform on practical limitations of hyperpolarized perfusion measurements, pivotal simulation parameters were varied over their respectively expected value ranges in vivo. Table 1 shows the parameters in the defined reference case (BASE) as well as their respective sweep ranges.

**Table 1:** Parameters investigated by simulations. BASE values refer to the respective parameter value used for the reference simulation. Range refers to the respectively investigated value range for each parameter

Parameter	BASE value	Range [min, max]	Step size
MBF [mL/min/g]	2.5	[0.5, 5.0]	0.5
B_0_ in-plane [ppm]	0.25	[0.0, 2.0]	0.5
B_dz_ through-plane [ppm]	0.0	[0.0, 1.0]	0.5
k_PL_ [s^−1^]	0.0	[0.0, 0.2]	0.05
v_max_ [m/s]	0.35	[0.175, 0.7]	0.175

Static parameters were set to: α = 60°, v_enc_ = 0.35 m/s, *w* = 0.2 m. T_1_/T_2_ values for pyruvate, lactate and bicarbonate were set to 30/0.1 s, 20/0.1 s and 20/0.15 s, respectively. T1 values were chosen based on separate experiments outlined below. In absence of available T2 values for cardiac tissue, conservative estimations were used based on hepatic tissue in rats [29]. For each simulation according to Equation (1) and Table 1, a second simulation was performed with a constant flip angle of 5° to assess the parameter impact on AIF measurements in vivo.

### Simulation post-processing

A total of 8 signal-to-noise ratio (SNR) levels were simulated: 5, 7.5, 10, 15, 20, 30, 40, 50. For each SNR level 20 realizations of noise were computed with respect to mean peak myocardial signal. Signal curves for the six AHA sectors and the AIF were then extracted, corrected for T_1_ relaxation and baseline corrected. AIF signals were scaled to compensate for the flip angle difference. For all myocardial sectors, the area-under-the-curve (AUC) and upslope parameters were calculated. The AUC was calculated by signal integration from AIF peak to myocardial peak, whereas upslope was determined by fitting a linear slope over 5 time points centered between AIF peak and myocardial peak signal.

MBF quantification was performed by iterative fitting of the IRF in Equation (2) for varying time shifts as proposed by Wissmann et al. [30]. Prior to quantification, a three-parameter gamma variate function was fitted to the AIF signal according to Equation (8) to compensate for the insufficient sampling rate and to reflect the treatment of in vivo data described in the data post-processing section. Absolute MBF was then calculated from fitted AIF signals and up-sampled myocardial signals as described below for in vivo data post-processing.

### Hyperpolarization and in vivo CMR

In vivo measurements were performed on a clinical 3 T wide-bore scanner (Ingenia, Philips Healthcare, Best, The Netherlands) equipped with a gradient system delivering 30 mT/m maximum amplitude at 195 T/m/s slew rate. A custom coil array with 6 ^13^C and 2 ^1^H receive channels was used for signal reception (Rapid Biomedical, Rimpar, Germany). Animals were placed in right recumbency inside the scanner and an electrocardiogram (ECG) unit was used for cardiac synchronization.

Samples of 0.75 mL neat pyruvic acid were doped with 15 mM AH111501 trityl radical and polarized for 3.5 h in a commercial 5 T SpinLab Polarizer (General Electric Healthcare, Waukesha, Wisconsin, USA) before dissolution in a buffer of 25 mL 0.1% EDTA water solution. Upon sample collection, the prepared solution was neutralized and diluted with 10.85 g of 0.72 M NaOH solution and 4.5 mL buffer solution at 0 °C to achieve a final injection medium with a pH value of 7 at body temperature. 25 mL of the final 300 mM ^13^C pyruvate solution were bolus injected over 2 s through femoral venous catheters 20 s after dissolution. Polarization levels and T_1_ relaxation times of the neat solution inside the 3 T magnetic field were established in separate experiments as 54 ± 3% and 71 ± 3 s, respectively. In vivo T_1_ relaxation times were determined to 32 ± 3 s, based on 3 measurements of hyperpolarized pyruvate solution diluted in porcine blood at a ratio of 1:10.

### Animal handling

Four healthy female swine (Edelschwein, weight 30–35 kg) were used for the experiments. After induction of general anesthesia, all swine were intubated and sheaths (5 F) were introduced into both femoral arteries and veins. 100 IU/kg unfractionated heparin was given intravenously and repeated every hour. General anesthesia was maintained with isoflurane (2–3%) by positive pressure ventilation with 100% oxygen. Heart rate, rhythm and variability, inspiratory and expiratory gases (CO_2_, O_2_, isoflurane), pulse oximetry, temperature, direct arterial blood pressure, urine output, and arterial and venous blood gases were monitored throughout the procedure. Cardiac stress was pharmacologically induced by intravenous administration of dobutamine (Dobutrex, TEVA Pharma AG, Basel, Switzerland) at increasing infusion rates until a heart rate of 120 bpm was reached (from a baseline heart rate between 70 and 85 bpm). Upon reaching the target heart rate, the dobutamine infusion rate was maintained during all imaging experiments. Three animals received a second injection of hyperpolarized pyruvate to assess reproducibility. After the procedure, all animals were euthanized in deep anesthesia by lethal injection of pentobarbital.

### CMR imaging

Dynamic series of ^13^C perfusion images in late systole were acquired with electrocardiogram (ECG) triggering starting five heartbeats after the start of injection to minimize bolus saturation in the RV blood pool. Ventilation was suspended for the first 45 s of imaging to avoid misregistration between individual time frames. Velocity selective excitation [11] was followed by an EPI readout with parameters: FOV = 120 × 120 mm^2^, slice thickness 15 mm, in-plane resolution 3.0 × 3.0 mm^2^, TE = 13.5 ms, TR = 1 heartbeat, FA: 60°, partial Fourier factor 0.65, readout duration 32 ms. An additional image stack in an apically adjacent slice with conventional excitation (FA: 5°) was interleaved in diastole of the same heart beat to provide AIF signal curves for absolute MBF quantification.

All in vivo images were reconstructed from raw data using MRecon (GyroTools LLC, Zurich, Switzerland) and zero-filled to a common resolution of 1 × 1 mm^2^. ^1^H images were then rotated, aligned and cropped to the FOV of the ^13^C images. Nyquist-ghosts of the EPI readout were removed by first order phase correction maximizing signal intensity in a predefined region of interest (e.g. LV blood pool). ^13^C coil combination was implemented as root of weighted sum of squares [31].

### Data post-processing

LV myocardium and LV blood pool were manually segmented on overlays of ^13^C perfusion and ^1^H reference images. The myocardium was subsequently divided into six segments corresponding to the basal / mid-ventricular slices in the 16 segment AHA model [27].

Magnitude coil sensitivities were estimated by fitting a plane over the entire FOV, using only regions with myocardial signal. A time point 3 s after myocardial bolus passage was selected to prevent overfitting of the sensitivities to local perfusion deficits. The resulting sensitivity plane was then visually inspected for plausibility and used to correct signal intensities in all previous image frames. [1-^13^C] pyruvate signal intensities were calculated as mean values over each segment in the dynamic image series, as well as the LV blood pool from the AIF scan (scaled to compensate for different flip angles). Myocardial signals were corrected for T_1_ relaxation (T_1_ = 32 s) and cropped 1 s after myocardial peak signal for quantification. Baseline offsets were corrected for by subtraction of mean noise levels prior to bolus arrival.

Three-parameter gamma-variate functions

8 $$ s(t)={at}^b{e}^{-\frac{t}{c}} $$

were fitted to the measured time curves *s*(*t*) for further analysis. Area under the curve (AUC) and up-slope were extracted from the fitted curves as semi-quantitative perfusion measures [3]. SNR / CNR was calculated as mean signal intensities divided by the standard deviation over a noise frame after ^13^C signal decay [32].

Absolute MBF quantification of in vivo data was performed by iterative fitting of a Fermi function as the impulse response for varying time shifts between 0 and 5 s. The first point in the time domain representation of the solution with the smallest fitting error was taken as the absolute MBF value and scaled to units of mL/min/g.

### ^1^H gadolinium DCE imaging

DCE perfusion CMR image series using ^1^H gadolinium were acquired in three of the four subjects for preliminary validation of the proposed method. Measurements were performed within 5 min after the last [1-^13^C] pyruvate injection. Between the ^13^C and ^1^H measurements, dobutamine stress CMR was maintained constant. To facilitate MBF quantification, a dual-bolus approach with 0.025 mmol/kg and 0.075 mmol/kg gadolinium (Gadovist 1.0, Bayer Healthcare, Berlin, Germany) was chosen [33]. Dynamic image series were acquired every heart beat using a saturation-recovery spoiled gradient echo sequence with parameters: FOV = 220 × 220 mm2,slice thickness 10 mm, in-plane resolution 3.0 × 3.0 mm2, TR = 1.9 ms, TE = 0.7 ms, FA = 15°, WET saturation [34] delay = 80–100 ms. A total of 100 dynamics were acquired during suspended ventilation. Post-processing, extraction of semi-quantitative perfusion indices and absolute MBF quantification was performed analogous to the ^13^C data.

## Results

### Simulations

**Fig. 2:**
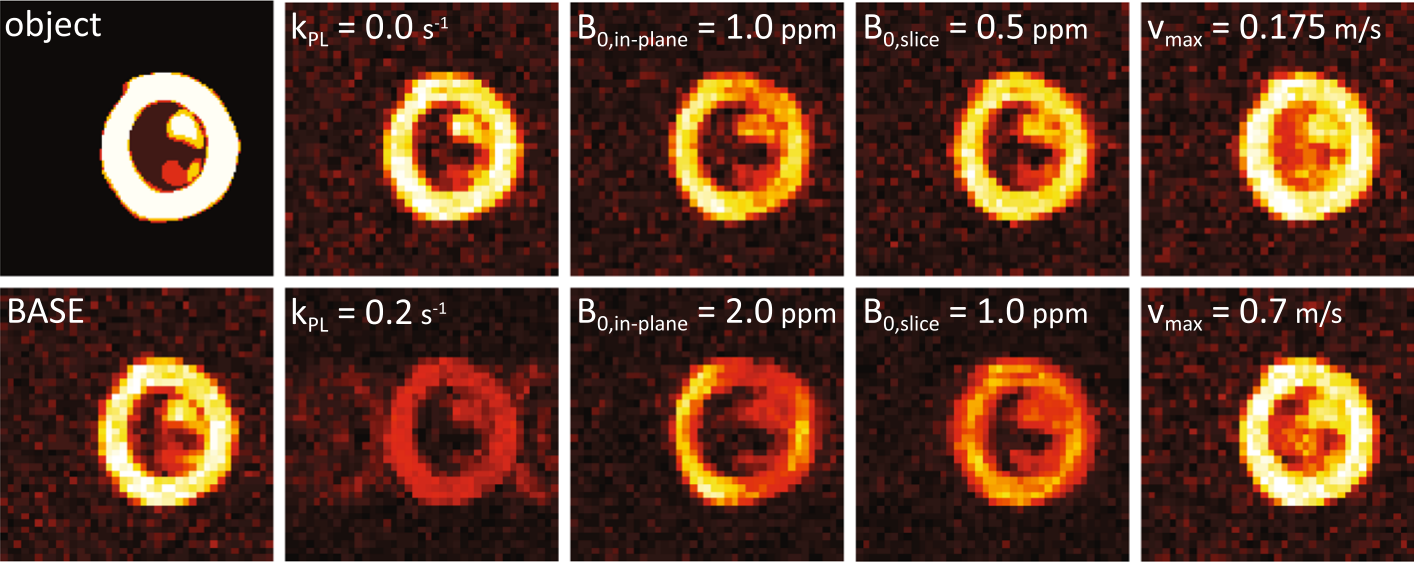
Simulated perfusion images at myocardial signal peak for signal-to-noise ratio (SNR) = 15. Effects of limited sampling resolution and variation of simulation parameters are illustrated. BASE case simulation values were: myocardial blood flow (MBF) = 2.5 mL/g/min, B_0_ in-plane = 0.25 ppm, B_0_ through-plane = 0.0 ppm, k_PL_ = 0.0 s^− 1^, v_max_ = 0.35 m/s., flip-angle = 60°, in-plane resolutio*n* = 3 × 3 mm^2^, field-of-view 120 × 120 mm^2^

Figure 2 qualitatively illustrates the effects of limited sampling resolution as well as variations of key parameters. Increased metabolic conversion results in reduced myocardial [1-^13^C] pyruvate signal with chemically shifted contamination from [^13^C] bicarbonate and [1-^13^C] lactate. In-plane B_0_ inhomogeneities give rise to EPI related geometrical distortions as well as signal loss due to intra-voxel dephasing. Through-plane B_0_ gradients retain geometrical accuracy but contribute to dephasing and signal loss. Deviations between encoding and actual blood velocities result in reduced blood pool suppression and partial volume effects.

**Fig. 3:**
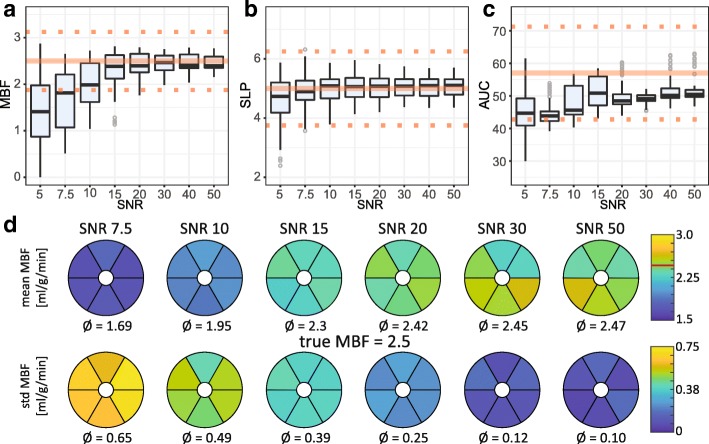
Simulation results for BASE as function of eight SNR levels. **a-c** Variability of absolute and semi-quantitative perfusion measures over myocardial sectors and 20 noise realizations. Reference values from input concentration dynamics are indicated by the solid line. Dotted lines indicate the ±25% interval around the reference value. **a** Absolute myocardial blood flow (MBF) in [mL / g / min]. For SNR ≥ 20 the values are confined to the ±25% interval. **b** Myocardial up-slope (SLP). Above SNR = 10 no further improvement of accuracy is apparent. **c** Area under the curve (AUC). **d** Mean and standard deviation of absolute MBF quantification over 20 noise realizations per myocardial sector

SNR dependency of absolute and semi-quantitative perfusion measures are illustrated in Fig. 3 for the BASE case. SNR levels ≥15 appear sufficient for absolute MBF quantification within a 25% uncertainty window. A systematic under-estimation towards lower SNR levels is apparent. Upslope appears largely insensitive to SNR variations with accurate results at SNR ≥ 7.5. AUC values exhibit systematic underestimation of ≥10% with outliers even at high SNR levels.

**Fig. 4:**
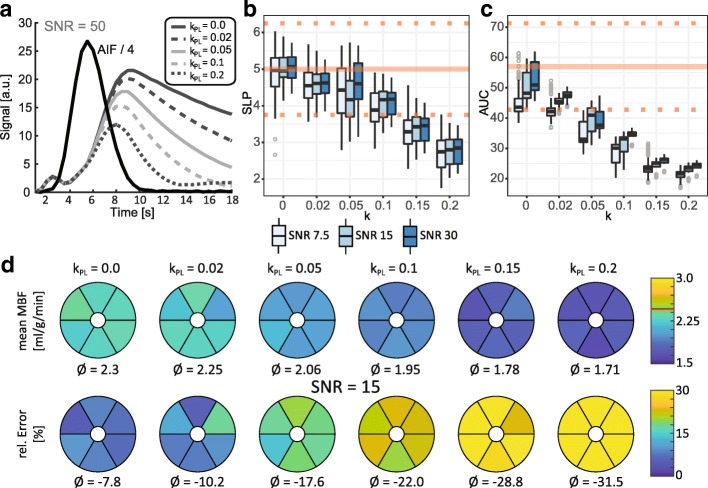
Simulation results for variations of kinetic conversion rate parameter k for SNR = 7.5, 15 and 30. Reference values from input concentration dynamics are indicated by the solid line. Dotted lines indicate the ±25% interval around the reference value. **a** Illustration of changes in myocardial response curve with increasing k values. Faster conversion at higher values results in a reduced pyruvate signal. Downstream lactate and Bicarbonate signal can cause signal contamination as illustrated in Fig. 2. **b-c** Semi-quantitative perfusion measures as function of k values analogous to Fig. 3. **b** Myocardial upslope (SLP). For k values ≥0.05 the SLP index is noticeably affected. **c** Area under the curve (AUC). **d** Mean absolute MBF quantification with relative error per myocardial sector for SNR = 15. Increasing conversion rates cause systematic underestimation of MBF. For values ≤0.1 the error is below 20%

Metabolic conversion is analyzed in Fig. 4. Increasing kinetic rates k_PL_ result in a reduced myocardial response signal. For k_PL_ ≤ 0.05 s^− 1^ the up-slope remains within 25% of the reference value and the relative error of absolute MBF quantification is ≤20%. AUC appears to be linearly dependent on k_PL_ and more strongly affected by increased metabolic conversion than the upslope parameter.

**Fig. 5:**
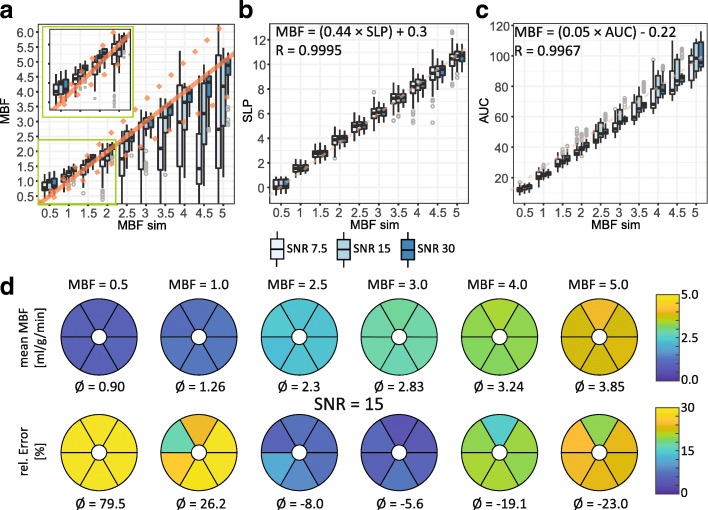
Simulation results for variations of myocardial blood flow (MBF) in units of mL/g/min for SNR = 7.5, 15 and 30. **a** Calculated MBF after reconstruction vs. simulated MBF value. Reference values used to calculate input concentration dynamics are indicated by the solid line. Dotted lines indicate the ±25% interval around the reference value. Simulated MBF values ≤1.0 result in overestimation for all SNR levels, whereas higher simulated MBF values are only underestimated for lower SNR levels. **b-c** Semi-quantitative perfusion measures as function of absolute MBF values analogous to Fig. 3. The dotted line represents the linear regression model calculated at SNR = 50 (top left corner). **b** Myocardial upslope (SLP). **c** Area under the curve (AUC). **d** Mean absolute MBF quantification with relative error per myocardial sector for SNR = 15. Strong overestimation at low MBF values, and moderate underestimation at high MBF values is apparent

Figure 5 shows the results obtained for simulated absolute MBF values between 0.5 and 5.0 mL /min/g. Low simulated MBF values between 0.5 and 1.0 mL/g/min lead to an SNR independent overestimation of 80 and 26% respectively. At higher simulated MBF values ≥3.5 mL/g/min underestimation for SNR levels < 30 is apparent. Semi-quantitative perfusion indices upslope and AUC present excellent linear dependency on simulated MBF with little SNR dependent variation. The respective linear regression models were calculated as MBF = 0.44 * upslope + 0.30 for upslope-to-MBF conversion (*R* = 0.999 at SNR = 50) and *MBF* = 0.053 ⋅ *AUC* − 0.224 for AUC-to-MBF conversion (R = 0.999 at SNR = 50).

**Fig. 6:**
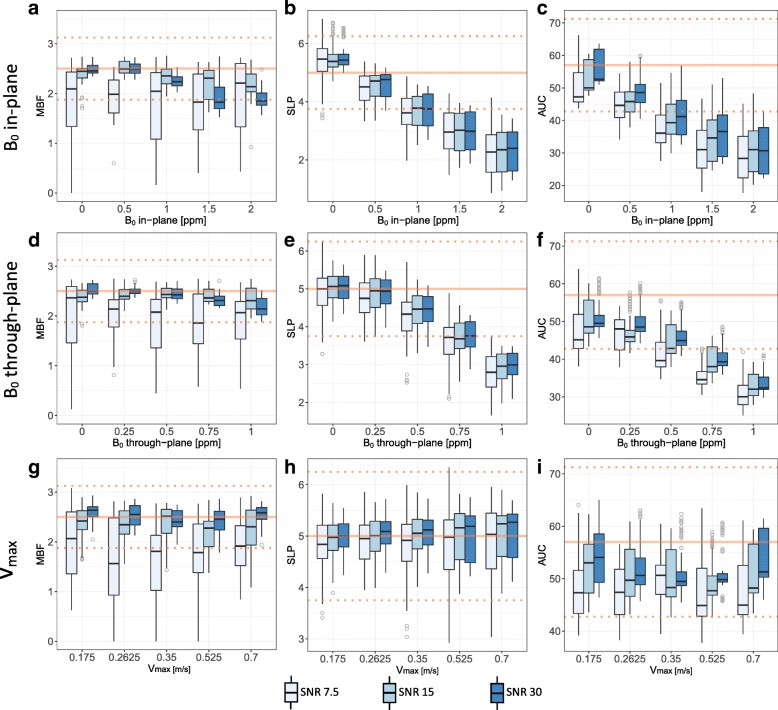
Simulation results for parameter variations with respective impact on absolute and semi-quantitative perfusion indices: **a-c** maximum B_0_ inhomogeneity in-plane. **d-f** B_0_ inhomogeneity through-plane. **g-i** maximum blood velocity inside the LV. Whereas B_0_ inhomogeneities larger than 0.5 ppm in either direction appear to have noticeable impact on absolute and semi-quantitative perfusion measures, no systematic dependency with respect to blood velocity is observed

Impact of B_0_ inhomogeneities and blood pool velocities is illustrated in Fig. 6. B_0_ inhomogeneities show similar impact for in-plane and through-plane directions: Up to 0.5 ppm, mean absolute and semi-quantitative perfusion measures are within 25% of the respective reference values for SNR ≥ 15. For stronger field gradients, absolute MBF quantification appears to be more robust than semi-quantitative measures. Misadjusted velocity encoding has little effect on the analysis of perfusion indices in the simulated case.

### In vivo

**Fig. 7:**
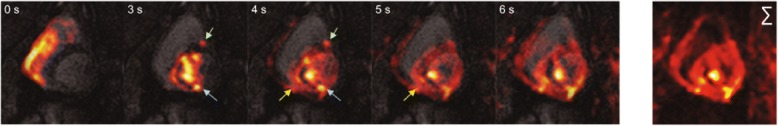
(**left**) Exemplary dynamic perfusion image series acquired in vivo. Myocardial bolus peak occurs between 4 and 6 s after start of acquisition. Arrows indicate the left anterior descending artery (LAD) (green), left circumflex (LCx) (blue) and right coronary artery (RCA) (yellow). (**right**) Myocardial bolus peak image summed over two frames. Enhanced signal in the lateral inferior segment is caused by signal leakage from the LCx and signal pile-up stemming from geometric distortions in the EPI image due to local off-resonances

An example of a dynamic perfusion image series is shown in Fig. 7. Myocardial peak signal is observed 5–6 s after start of the scan (7–8 s after start of injection). The three main cardiac arteries (left anterior descending (LAD), left circumflex (LCx), right coronary artery (RCA)) are visible after 4 s. Summation over two time frames at myocardial peak signal is shown for qualitative assessment of perfusion. The LV and RV myocardium is clearly visible. Arterial signal leads to local hyper-intensities around the respective vessels.

**Fig. 8:**
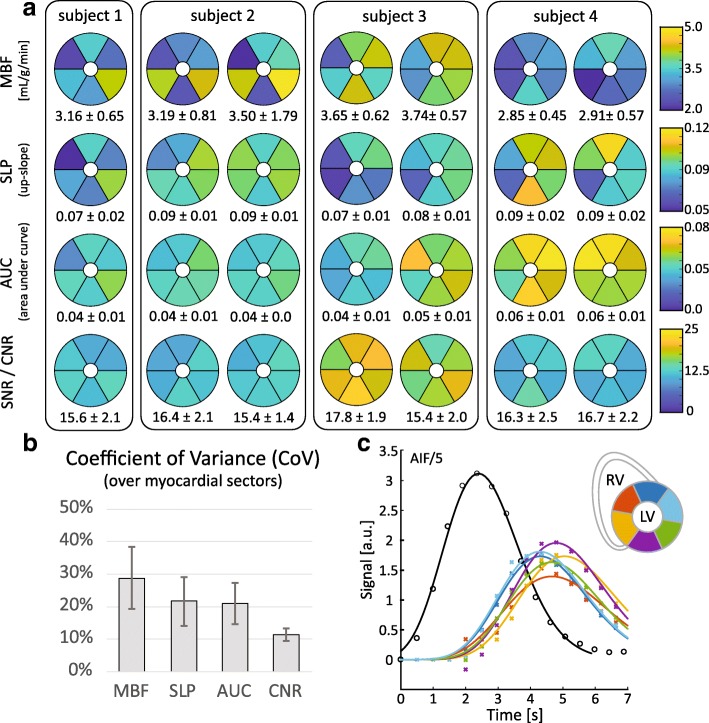
Perfusion indices analysed in 5 measurements in 4 swine. Repeat measurements were performed in three swine. **a** Sector wise absolute and semi-quantitative perfusion indices and SNR for each measurement. Mean and standard deviation over all sectors are presented below the respective maps. **b** Mean intra-myocardial Coefficient of Variance over all measurements for each perfusion index. Error bars indicate the respective standard deviations. **c** Exemplary perfusion curves acquired in subject 3. Raw data points are depicted with markers; solid lines show the respective fitted gamma-variate functions

Figure 8 shows the absolute and semi-quantitative perfusion analysis for seven stress measurements in 4 swine. Under in vivo conditions, the myocardial SNR across the myocardium ranges from 11.0 ± 1.4 to 19.3 ± 2.1. Absolute MBF quantification yielded values between 2.85 ± 0.45 and 3.74 ± 0.75 mL/g/min, with mean variations ≤10% in repeat measurements. Normalized upslope values ranged from 0.07 ± 0.02 to 0.09 ± 0.1 (mean intra-subject variation ≤15%), and normalized AUC values from 0.04 ± 0.01 to 0.06 ± 0.01 (mean intra-subject variation ≤40%). Intra-myocardial coefficients of variance for MBF, upslope, AUC and CNR were determined as 29 ± 19%, 22 ± 15%, 21 ± 13% and 11 ± 4%, respectively.

**Fig. 9:**
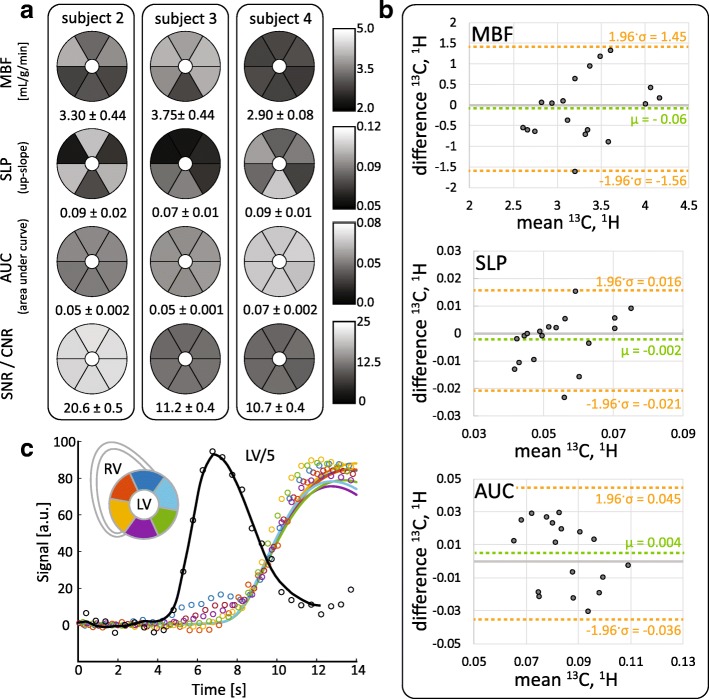
Summary of preliminary ^1^H gadolinium DCE validation data acquired in n = 3 subjects under the same stress condition as the ^13^C measurements. **a** Sector wise absolute and semi-quantitative perfusion indices and SNR for each ^1^H DCE measurement. Mean and standard deviation over all sectors are presented below the respective maps. **b** Comparison of sector-wise perfusion indices derived from dual-bolus ^1^H gadolinium DCE measurements and hyperpolarized ^13^C pyruvate measurements. **c** Exemplary perfusion curves acquired in subject 3. Raw data points are depicted with markers; solid lines show the calculated tissue response for the fitted Fermi function

Figure 9 shows preliminary validation data obtained from ^1^H gadolinium DCE imaging in three of the four subjects. Absolute MBF quantification yielded values between 2.90 ± 0.08 and 3.75 ± 0.44 mL/g/min, with myocardial SNR values ranging from 10.7 ± 0.4 to 20.6 ± 0.5. Bland-Altman analysis revealed good agreement between quantitative perfusion measures derived from ^13^C and ^1^H DCE image series.

## Discussion

In this study, we employed extensive simulations to assess experimental requirements and limitations for absolute and semi-quantitative myocardial CMR perfusion imaging using hyperpolarized [1-^13^C] pyruvate. We have demonstrated that for rapid bolus injections, the metabolic conversion into [1-^13^C] lactate and [^13^C] bicarbonate can largely be neglected as the information related to perfusion is contained in a very short window of a few seconds. In vivo stress measurements in swine suggest feasibility for the obtainable SNR and expected parameter range with respect to metabolic kinetics and B_0_ inhomogeneities.

Metabolic conversion of hyperpolarized [1-^13^C] pyruvate into the downstream metabolites [1-^13^C] lactate, [^13^C] bicarbonate and [1-^13^C] alanine is a potential hindrance to accurate perfusion assessment. The presented simulations, however, indicate that the myocardial response signal is only weakly affected for rate constants k_PL_ < 0.1 s^− 1^. Based on measurements in rat hearts [20, 21], skeletal muscle [35] and tumor cell cultures [36] the expected in vivo values for k_PL_ are in the range of 0.01 s^− 1^ to 0.1 s^− 1^. Absolute MBF quantification and the upslope perfusion index generally appear more robust towards metabolic conversion than the AUC index. In order to improve the robustness over a wider range of kinetic rate constants or to allow slower bolus injection, combining the chemically shifted signals of individual metabolites is envisioned.

As described previously, the velocity-selective excitation employed in this study is tailored for measurements under stress condition with increased heart rates, contractility and therefore enhanced blood velocities inside the LV blood pool [11]. This potentially limits applicability in patient cohorts that cannot tolerate dobutamine infusion. Diagnostically, however, quantitative measurements under stress are considered of prime diagnostic value as early stage perfusion deficits only present at maximum workload/vasodilation [37]. A similar workload dependency can also be expected for the onset of metabolic alterations.

B_0_ inhomogeneities are a common problem in hyperpolarized imaging, since relatively long readout trajectories such as EPI are used. The lower gyromagnetic ratio of the ^13^C nucleus results in comparatively long readouts with low bandwidth in phase encoding direction. Phase offsets from B_0_ inhomogeneities, as well as motion, can cause significant signal loss due to dephasing and geometric distortions. Different approaches for geometric distortion correction for hyperpolarized EPI acquisitions have been proposed. Inverting the readout direction in additional echoes [38] or by alternating the blip direction [39] enables the calculation of distortion maps for correction. These methods however require tailored pulse sequences and full k-space sampling, as well as accurate image-based registration of distorted images for deriving a B_0_ map estimate. Without distortion correction, the presented simulations indicate underestimations up to 30% on semi-quantitative perfusion indices for typically achievable B_0_ variations of < 1.0 ppm over the imaging volume. Absolute MBF quantification appears more robust towards B_0_ inhomogeneities at higher SNR levels with respect to mean values. However, larger B_0_ inhomogeneities give rise to pronounced signal variations over the myocardium which potentially limits the diagnostic value. The observed robustness towards B_0_ inhomogeneities in simulations is therefore contingent on similar signal dephasing effects inside the AIF and myocardial compartments. Under in vivo conditions fast flowing blood near the aortic valve (aortic jet) and strong off-resonances in the proximity of venous vessels can cause signal pile-up or dephasing in the septal and lateral inferior sectors respectively, which results in larger intra-myocardial variability. Additionally, phase accrual due to blood flow can significantly dampen the apparent amplitude of the AIF signal and result in underestimations of absolute MBF. Fat shift direction, phase encoding direction and the trigger delay need therefore be considered carefully during planning in vivo to avoid unnecessary artifacts.

Comparison of absolute and semi-quantitative perfusion measures in simulations revealed excellent linear relationship between MBF and upslope and AUC, respectively. This confirms previous findings based on conventional contrast agents that upslope [40, 41] and AUC [42] are suitable surrogate indices to assess MBF and perfusion reserve. Based on simulation results, the upslope index appears superior to AUC for hyperpolarized [1-^13^C] pyruvate, due to lower parameter dependency overall and less bias introduced by baseline correction steps. Of all the derived perfusion indices, MBF showed the strongest SNR dependent variability, especially for higher MBF values > 4 mL/g/min. These findings suggest that in vivo measurements obtained in this range are only accurate for relatively high SNR values. The practically reliable measurement range for MBF quantification is therefore subject to the achievable SNR with a given experimental setup.

Using a plane fit for the correction of spatial variance in receive coil sensitivity inherently assumes homogeneous signal intensities over the region of interest. By choosing a time frame after initial myocardial bolus passage, the effect of impaired perfusion is reduced, as hypo-perfused areas are characterized by delayed contrast agent uptake. Estimated sensitivity maps were visually inspected to ensure that the resulting sensitivity gradient was plausible with respect to coil placement. For clinical applications with possibly vast perfusion deficits, a different sensitivity correction strategy might be required.

Although the presented simulation framework reflects image acquisition more accurately than previous work [43], acquisitions in vivo are aggravated by additional effects: Firstly, cardiac motion and hemodynamics can introduce additional dephasing and distortions as well as misregistration between image frames. Hemodynamics are particularly important for hyperpolarized contrast agents, as each excitation of the imaging slice saturates part of the contrast agent bolus magnetization in the blood compartment. In the presented simulation, this effect was neglected as the dynamics of bolus localization and concentration are not available and would require extensive 3D computational fluid dynamics. Improper adjustment of the velocity selective excitation may therefore have stronger impact on perfusion measurements in vivo than indicated by simulations. Other common obstacles in hyperpolarized imaging in general, such as coil combination strategies at low SNR levels without measured sensitivity maps and flip-angle miscalibration were also omitted in this study.

Feasibility of cardiac perfusion CMR with interleaved AIF sampling using hyperpolarized [1-^13^C] pyruvate was demonstrated in vivo in swine. As predicted by simulations, the achievable SNR, B_0_ homogeneity and expected kinetic conversion rates appear feasible for absolute and semi-quantitative perfusion assessment. Overall data quality was comparable to a previous study using hyperpolarized [^13^C] urea [11]. Localized phase variations caused by the aortic jet resulted in geometric distortion and signal pile-up artifacts in the septal region which compromised accurate analysis in this sector. Optimizing the readout strategy is believed to be crucial in improving overall quality of hyperpolarized perfusion CMR. The phase sensitivity of EPI readouts could be reduced by increasing the bandwidth along the phase encoding direction if higher gradient system performance becomes available. Similarly, spiral readouts that are inherently insensitive to flow artifacts require gradient specifications beyond most current clinical systems in order to achieve sufficient resolution. Flow-compensated EPI [44] could minimize motion induced artifacts, yet results in prohibitively long readout durations and echo times.

Full validation regarding the accuracy of quantitative perfusion CMR using hyperpolarized pyruvate with a gold-standard method remains to be performed. However, preliminary ^1^H gadolinium DCE data obtained from the same subjects under maintained stress condition have been processed and are presented in Fig. 9. Good agreement between the two modalities with respect to mean values is found, variability is however relatively high in both cases due to current experimental limitations. Our current combined ^13^C TxRx/^1^H Rx coil setup is limited in particular regarding ^1^H sensitivity which in turn compromised the quality of ^1^H perfusion data. In order to acquire state-of-the-art ^1^H images during the same stress protocol, future validation studies need to improve the experimental setup with respect to ^1^H receive coil architecture.

Although adenosine is often used as a vasodilator in clinical stress CMR, administration protocols in swine are still being established. The required adenosine doses are significantly higher compared to humnan protocols and cause systemic vasodilation and hypotension in the porcine model [45]. These systemic side-effects warrant additional pharmacological intervention, e.g. with α_1_-adrenergic receptor agonists [46, 47]. In this proof-of-principle study, dobutamine was hence used for the ease of inducing a strong vasodilatory response [48, 49]. Accordingly, the ^1^H perfusion protocols had to be adjusted to cope with high heart rates during dobutamine stress CMR, which also required compromises regarding the optimal saturation delay in the ^1^H perfusion sequence. Upon translation of our work to humans, adenosine will be the preferred stressor and hence clinically tested ^1^H perfusion protocols are applicable.

The quantification methods used for ^13^C pyruvate data in this study have been directly adopted from existing methods for ^1^H gadolinium enhanced perfusion stress CMR. Although initial comparison between both modalities looks promising, further work is required in the development of tailored perfusion models for ^13^C pyruvate. Such improved models should not only account for differences in molecular size and therefore permeability, but also entail active transport over cell membranes and possible metabolic conversion.

## Conclusion

In this study, a comprehensive simulation framework to assess requirements for accurate absolute and semi-quantitative cardiac perfusion CMR using hyperpolarized [1-^13^C] pyruvate has been presented along with demonstration of in vivo feasibility. With the current translation of dissolution dynamic nuclear polarization into humans, the assessment of myocardial perfusion CMR with hyperpolarized [1-^13^C] pyruvate holds potential for the diagnosis of coronary artery and microvascular disease.

## Abbreviations

AHA: American Heart Association (segmentation)
AIF: Arterial input function
AUC: Area under the curve
CMR: Cardiovascular magnetic resonance
CNR: Contrast-to-noise ratio
CoV: Coefficient of variance
DCE: Dynamic contrast enhancement
ECG: Electrocardiogram
EPI: Echo planar imaging
FA: Flip angle
FOV: Field-of-view
IRF: Impulse response function
LAD: Left anterior descending coronary artery
LCx: Left circumflex coronary artery
LV: Left ventricle/left ventricular
MBF: Myocardial blood flow
RCA: Right coronary artery
RF: Radio-Frequency
RV: Right ventricle/right Ventricular
SNR: Signal-to-noise ratio
TE: Echo time
TR: Repetition time
